# Adult Diagnosis of Infantile Hemangioma with Minimal or Arrested Growth of the Heel: A Case report

**DOI:** 10.1159/cde/abvag008

**Published:** 2026-09-30

**Authors:** Mohammed Shanshal, Emily Moon, Georgios Kallikas

**Affiliations:** a Department of Dermatology, Imperial College Healthcare NHS Trust, London, UK; b Imperial College London, London, UK; c Department of Histopathology, Imperial College Healthcare NHS Trust, London, UK

**Keywords:** Infantile hemangioma with minimal or arrested growth, Abortive hemangioma, Case report

## Abstract

**Introduction:**

Infantile hemangioma with minimal or arrested growth (IH-MAG) is a recognized subtype of infantile hemangioma that presents as a predominantly flat telangiectatic or reticular lesion with little or no postnatal proliferation and may be mistaken for capillary malformation or other superficial vascular anomalies.

**Case Presentation:**

We report a 22-year-old woman with a long-standing asymptomatic reticular vascular patch on the posterolateral heel, reported by family to have been present since infancy and stable over time. Punch biopsy showed a predominantly fibrosed dermis containing sparse small vascular profiles, while GLUT1 immunohistochemistry highlighted lesional endothelial staining. Taken together, the clinicopathologic findings supported infantile hemangioma lineage and favored IH-MAG, likely reticular type.

**Conclusion:**

IH-MAG may persist into adulthood as a subtle acral lesion in which capillary malformation is the main clinical mimic. Accurate diagnosis, supported by clinicopathologic correlation, is clinically relevant because it identifies a lesion expected to remain stable and not require active intervention.

## Introduction

Infantile hemangioma with minimal or arrested growth (IH-MAG), also termed abortive infantile hemangioma, is a recognized subtype of infantile hemangioma in which the proliferative component involves 25% or less of the lesion surface area [1–3]. Unlike classic infantile hemangioma, these lesions are often apparent at or shortly after birth as flat telangiectatic, reticular, or stain-like patches, show little subsequent proliferation, and have a marked predilection for the lower body [1–3]. Because they may resemble capillary malformation, diagnosis can be delayed, particularly in clinically subtle lesions. Reticular distal lower limb phenotypes and acral involvement are well documented [4, 5]. Unlike classic infantile hemangioma, which typically undergoes rapid postnatal proliferation followed by gradual involution, IH-MAG shows little or no proliferative phase and may remain clinically stable or involute only subtly over time [1–3]. This distinction has practical relevance in adulthood, where recognition of IH-MAG can support reassurance and avoid unnecessary intervention once progressive vascular mimics have been excluded. This case report was prepared in accordance with the CARE guidelines, and the completed CARE checklist is provided as online supplementary material (for all online suppl. material, see https://doi.org/10.1159/cde/abvag001).

## Case Report

A 22-year-old woman presented with a long-standing, asymptomatic vascular patch on the posterolateral heel, reported by family to have been present since infancy and stable in size and contour. She was otherwise healthy, took no regular medications, and had no relevant past medical history. There was no personal or family history of vascular anomalies or other relevant dermatologic disease. Examination showed a faint, reticular brown-violaceous telangiectatic patch without nodularity or a palpable subcutaneous component (Fig. 1a). There was no history of ulceration, bleeding, or interval change.

**Fig. 1.**
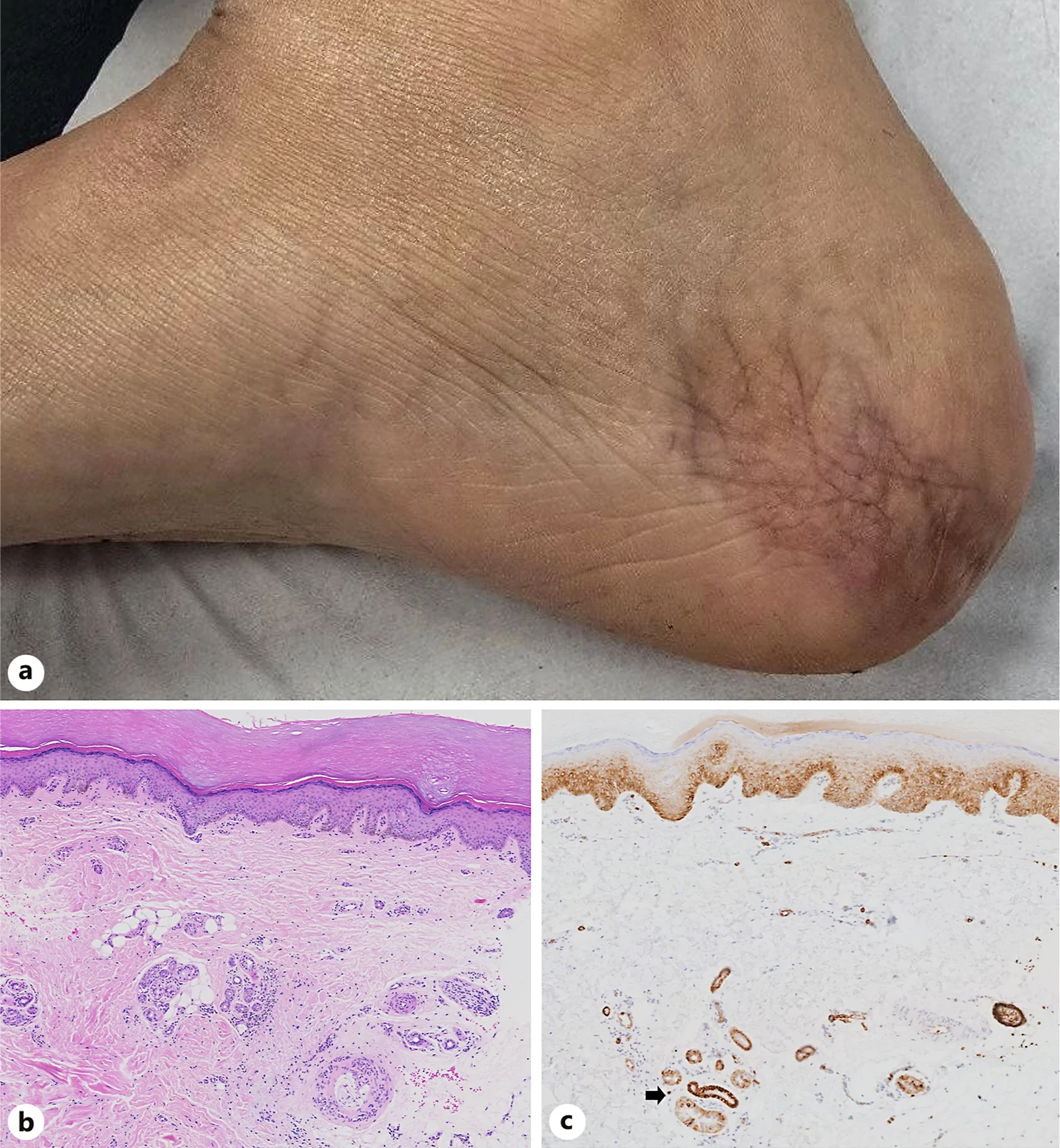
**a** Clinical photograph showing a faint, reticular violaceous telangiectatic patch on the posterolateral heel. **b** Hematoxylin and eosin section showing a predominantly fibrosed dermis containing sparse small-caliber vascular channels without a discrete lobular capillary proliferation (×5; Philips DigiPath). **c** GLUT1 immunohistochemistry showing strong epidermal staining as an internal positive control and endothelial staining in scattered dermal vascular profiles (×5; Philips DigiPath).

Because the lesion remained clinically unclassified in adulthood despite long-standing stability, and the patient sought diagnostic clarification, a punch biopsy was undertaken primarily to distinguish infantile hemangioma lineage from capillary malformation and related superficial vascular lesions. The biopsy demonstrated a predominantly fibrosed/sclerotic dermis containing sparse small vascular profiles, without a discrete lobular capillary proliferation in the sampled tissue (Fig. 1b). GLUT1 immunohistochemistry showed strong epidermal staining as an internal positive control and highlighted endothelial staining within scattered dermal vascular profiles (Fig. 1c). Taken together, the clinicopathologic findings supported infantile hemangioma lineage and favored IH-MAG, likely reticular type.

The principal diagnostic limitation was the absence of contemporaneous childhood photographs or medical documentation, so final classification relied on adult clinicopathologic correlation and the reported history of infantile onset. Nevertheless, the combination of a long-standing flat reticular acral lesion and endothelial GLUT1 positivity favored IH-MAG, likely reticular type, over capillary malformation. No active treatment or further follow-up was required, and the patient was reassured regarding the benign diagnosis and expected clinical stability of the lesion.

## Discussion

IH-MAG is best diagnosed by clinicopathologic correlation when the clinical morphology is subtle and routine histopathology is not classic [1, 2]. In this setting, GLUT1 immunoreactivity is particularly useful because it supports infantile hemangioma lineage in lesions that may otherwise resemble capillary malformation. Ma et al. [2] further highlighted the distinctive clinical, histopathologic, and immunohistochemical features of this underrecognized subset, and Oiso et al. [6] described a GLUT1-positive nonproliferative infantile hemangioma clinically mimicking port-wine stain.

Although GLUT1 immunoreactivity supports infantile hemangioma lineage, it should not be interpreted as diagnostic in isolation. Verrucous vascular lesions, including those historically termed verrucous hemangioma or verrucous venous malformation, represent the most relevant GLUT1-positive mimic in an acral context; however, this diagnosis was not supported clinically by the absence of a verrucous or hyperkeratotic surface, nor histologically by the absence of a verrucous vascular malformation pattern. Other GLUT1-positive vascular lesions reported in the literature, including angiokeratoma, epithelioid hemangioendothelioma, and angiosarcoma [7], were not favored because the lesion was flat, reticular, clinically stable since infancy, and lacked nodularity, ulceration, progressive change, or cytologic atypia. The diagnostic conclusion, therefore, rested on clinicopathologic correlation rather than GLUT1 positivity alone.

In the present case, the heel location and reticular morphology are compatible with the recognized phenotypic spectrum of IH-MAG [4, 5]. Bessis et al. [4] described reticular distal lower limb lesions, and Neri et al. [5] identified acral hand or foot involvement in 16 of 79 cases (20.3%). Although the biopsy did not show a classic lobular capillary proliferation, the combination of long-standing flat reticular acral morphology, reported infantile onset, and endothelial GLUT1 positivity favored IH-MAG, likely reticular type, over capillary malformation [2, 4–6]. Overall, the clinicopathologic findings were most consistent with IH-MAG of the heel, likely reticular type, with capillary malformation as the principal clinical mimic. In this context, establishing the correct diagnosis had a direct clinical consequence: reassurance and avoidance of unnecessary intervention.

## Statement of Ethics

Ethical approval was not required for this single-patient case report in accordance with local institutional policy. The patient provided written informed consent for publication of de-identified clinical details and images.

## Conflict of Interest Statement

The authors declare no conflict of interest.

## Funding Sources

No funding was received for this work.

## Author Contributions

Conceptualization, supervision, and writing – original draft: M. Shanshal. Investigation, data curation, and writing – review and editing: M. Shanshal, E. Moon, and G. Kallikas. Visualization: M. Shanshal and G. Kallikas. All authors approved the final manuscript.

## Data Availability

All data underlying this case report are included in the manuscript and figure. Further inquiries can be directed to the corresponding author.
